# Heat Stress Impedes Development and Lowers Fecundity of the Brown Planthopper *Nilaparvata lugens* (Stål)

**DOI:** 10.1371/journal.pone.0047413

**Published:** 2012-10-11

**Authors:** Jiranan Piyaphongkul, Jeremy Pritchard, Jeff Bale

**Affiliations:** School of Biosciences, University of Birmingham, Birmingham, West Midlands, United Kingdom; University of Lancaster, United Kingdom

## Abstract

This study investigated the effects of sub-lethal high temperatures on the development and reproduction of the brown plant hopper *Nilaparvata lugens* (Stål). When first instar nymphs were exposed at their ULT_50_ (41.8°C) mean development time to adult was increased in both males and females, from 15.2±0.3 and 18.2±0.3 days respectively in the control to 18.7±0.2 and 19±0.2 days in the treated insects. These differences in development arising from heat stress experienced in the first instar nymph did not persist into the adult stage (adult longevity of 23.5±1.1 and 24.4±1.1 days for treated males and females compared with 25.7±1.0 and 20.6±1.1 days in the control groups), although untreated males lived longer than untreated females. Total mean longevity was increased from 38.8±0.1 to 43.4±1.0 days in treated females, but male longevity was not affected (40.9±0.9 and 42.2±1.1 days respectively). When male and female first instar nymphs were exposed at their ULT_50_ of 41.8°C and allowed to mate on reaching adult, mean fecundity was reduced from 403.8±13.7 to 128.0±16.6 eggs per female in the treated insects. Following exposure of adult insects at their equivalent ULT_50_ (42.5°C), the three mating combinations of treated male x treated female, treated male x untreated female, and untreated male x treated female produced 169.3±14.7, 249.6±21.3 and 233.4±17.2 eggs per female respectively, all significantly lower than the control. Exposure of nymphs and adults at their respective ULT_50_ temperatures also significantly extended the time required for their progeny to complete egg development for all mating combinations compared with control. Overall, sub-lethal heat stress inhibited nymphal development, lowered fecundity and extended egg development time.

## Introduction

The effects of climate change on organisms and ecological communities are a highly topical issue. Insects are a taxon with limited ability to regulate their body temperature and are thus directly impacted by both prevailing weather and longer term climate change. Research on insect-climate interactions has focused on the measurement of thermal thresholds and lethal limits ([1], [2], [3], [4]), responses to manipulated conditions representing different scenarios of climate warming ([5], [6], [7], [8]) and shifts in distributions or changes in phenology detected through analyses of long term datasets ([9], [10], [11], [12], [13], [14], [15]). In general, more is known about the low temperature ecophysiology of insects ([16], [17], [18], [19], [20], [21], [22], [23]) than the effects of high temperatures, though upper thermal limits have been measured for a number of species ([24], [25], [26], [27], [28]). Also, whilst many studies have measured critical thermal thresholds at both low ([20], [23], [29], [30], [31]) and high temperatures ([2], [32], [33], [34], [35], [36]), less is known about the impacts of sub-lethal thermal stress on surviving individuals, though effects on development and reproduction have been reported ([37], [38], [39], [40], [41]). Climate change can affect terrestrial ectothermic species by modifying the structure of their physical environment, and by the associated changes in the thermal regime or temperature profile of the habitat ([42], [43], [44]). The mechanistic link between the biophysical environment and individual performance will directly affect demographic (e.g. survivorship, growth and reproduction) and population level phenomena (e.g. density and age structure) ([45]). Thus, a central issue in insect ecophysiology is how environmental factors such as temperature affect physiological performance ([1], [46], [47], [48], [49]). Temperature has a direct effect on the growth and development of insects ([50], [51], [52], [53], [54], [55]). The temperature-development relationship is approximately linear, increasing progressively to a maximum level beyond which the rate decreases and the response curve becomes markedly asymmetrical through the effects of heat stress and approaching lethality ([21], [56], [57], [58], [59], [60]). In addition, both longevity and fecundity of insects reach a maximum at species-specific optimum temperatures and more or less symmetrically decrease at both the lower and upper limits of tolerance ([61], [62]). Understanding the behavioural and physiological responses of insects to thermal stress will inform predictions about how climate warming could affect distributions, changes in pest status, and the likelihood of species extinctions ([63]). A number of studies have investigated the effects of temperature on development and fecundity e.g. *Nilparvata lugens* ([64], [65], [66], [67], [68], [69], [70]), small brown planthopper *Laodelphax striatellus* ([38], [71], [72]), the butterfly *Pararge aegeria* ([73]) and the pea leafminer *Liriomyza huidobrensis* [(74)].

The brown planthopper *Nilaparvata lugens* (Stål) (Order Hemiptera; Family Delphacidae) is the most serious rice pest in Asia, affecting a wide range of economically important rice crops that arose from the green revolution ([75], [76], [77], [78], [79]). *Nilaparvata lugens* is ‘sucking pest’ which removes sap from the xylem and phloem tissues of the rice stem ([80]). Severely damaged rice plants desiccate through the effects of feeding and ovipositor damage, a condition known as ‘hopper burn’ ([81]). *Nilparvata lugens* is also a vector of rice virus diseases, such as ‘grassy stunt’ ([75], [82], [83], [84]). *Nilaparvata lugens* populations fluctuate in response to changing environmental conditions, both physical (abiotic) and biotic, and can lead to pest outbreaks ([85]). In general, *N. lugens* is endemic to the Asian sub-tropical region, though its range can expand temporarily every summer as far north as Japan and Korea through long-distance migrations from the tropics ([75], [86]). As tropical species experience less seasonal variation in temperature they generally have narrower thermal tolerances compared with temperate species ([87], [88], [89]).

Much of the previous research on *N. lugens* has focused on the effect of rearing at different constant or variable temperatures on development and fecundity ([69], [90]) and on the impact of variation in the dietary composition of resistant cultivars on reproductive output ([68], [75], [91]). By comparison, the effects of sub-lethal heat stress on development and reproduction have received little attention but are likely to become more important in a scenario of climate warming. The mean summer day time high temperature in China varies from 37 to 41°C ([92]) and can rise to 50°C in some sub-tropical countries ([93]). Temperatures in this range are of interest because a recent study on *N. lugens* [28] found that nymphs were less heat tolerant than adults and concluded that in some parts of its distribution and under current climatic regimes, juvenile stages of *N. lugens* could become immobilised through heat stress and might be killed by high temperature exposure. However, even though insects may survive thermal stress, there may be sub-lethal effects on key processes that would impact negatively on population abundance, and hence the pest status of species such as the brown plant hopper. This raises the interesting question of whether insects living in tropical areas are sufficiently heat tolerant to survive under current conditions and if they can also adapt to the more stressful climatic regimes that may be experienced in the future.

Using knowledge gained on the upper lethal temperatures of nymphal and adult *N. lugens*, this study investigated the effects of sub-lethal high temperatures applied at different life cycle stages on the subsequent development, reproduction and longevity.

## Results

### Effect of Sub-lethal High Temperatures on Development and Longevity

When first instar nymphs were exposed at their ULT_50_ of 41.8°C mean times required to complete nymphal development increased from 15.2±0.3 (n = 31) and 18.2±0.2 (n = 19) days for male and female nymphs to 18.7±0.2 (n = 21) and 19.0±0.2 (n = 29) days respectively in the treated insects. Exposure at the first instar increased the longevity of adult females (from 20.6±1.1 to 24.4±1.0 days), but adult males were unaffected (longevity of 25.7±1.0 and 23.5±1.1 days for control and treated insects); however, mean development time of treated males was shorter than that for the control males. Mean total longevity was also increased in female insects (from 38.8±1.0 to 43.4±1.0 days), but the lifespan of male insects was similar between the control and treated males (40.9±0.9 and 42.2±1.1 days).

The increase in mean development time from nymph to adult after exposure at the ULT_50_ was significant (F_1, 96_ = 64.641, p<0.001), with a difference between the sexes (F_1, 96_ = 35.676, p<0.001) and in the interaction between the temperature treatment and sex (F_1, 96_ = 25.398, p<0.001). By comparison, there was no difference in adult longevity between the control and treated groups (F_1, 96_ = 0.525, p = 0.470), nor between the sexes (F_1, 96_ = 3.615, p = 0.060), but the interaction between the temperature treatment and sex was significant (F_1, 96_ = 7.342, p = 0.008). There was a significant effect of temperature on total longevity (F_1, 96_ = 8.764, p = 0.004), but no difference between the sexes (F_1, 96_ = 0.236, p = 0.628), nor in the interaction between the treatment and sex (F_1, 96_ = 2.645, p = 0.107).

**Figure 1:**
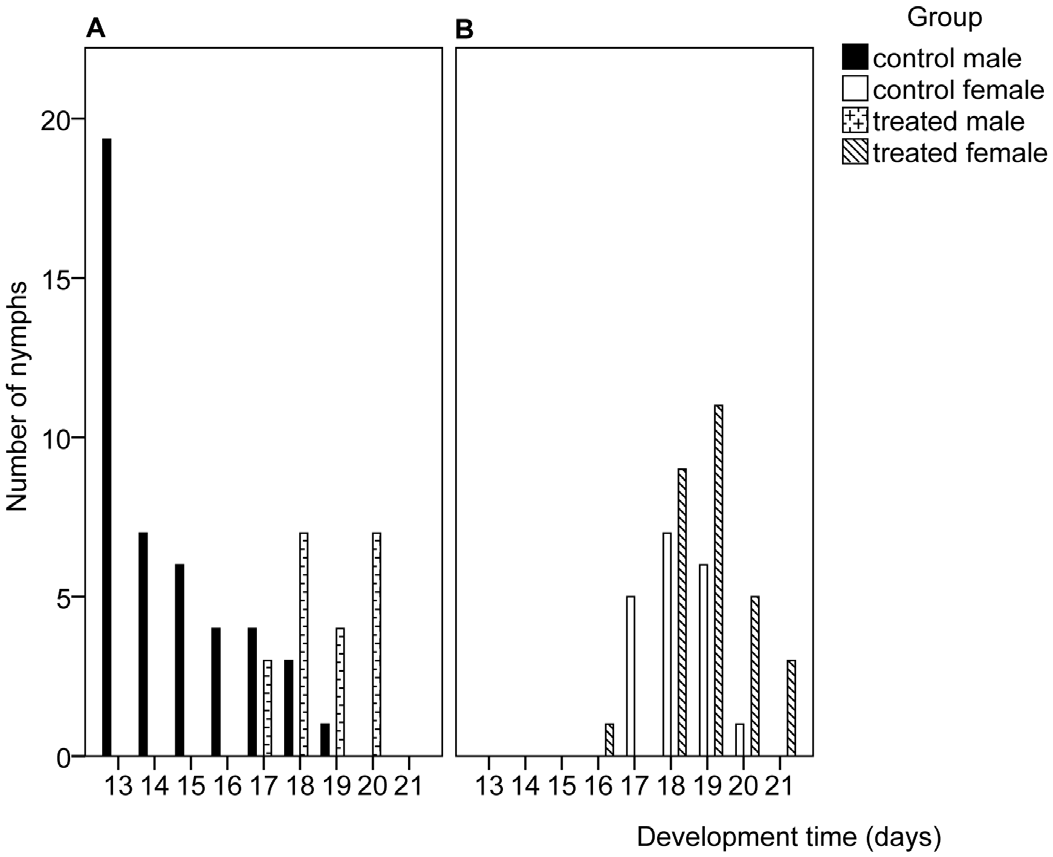
Range of development times for the nymphal stages of *Nilaparvata lugens* after exposure at the ULT_50_. N = 50 for control (31 male and 19 female) and treatment (21 male and 29 female) groups.

The range of times required for nymphs to complete development to adult is shown in Figure 1A and 1B. Whilst the overall range of treated males (17–20 days) and treated females (16–21 days) was similar to that of the control groups (13–19 days for males and 17–20 days for females), within these ranges, the treated insects generally took longer to complete nymphal development in both males (F_1, 50_ = 66.247, p<0.001) and females (F_1, 46_ = 6.959, p = 0.011).

**Figure 2:**
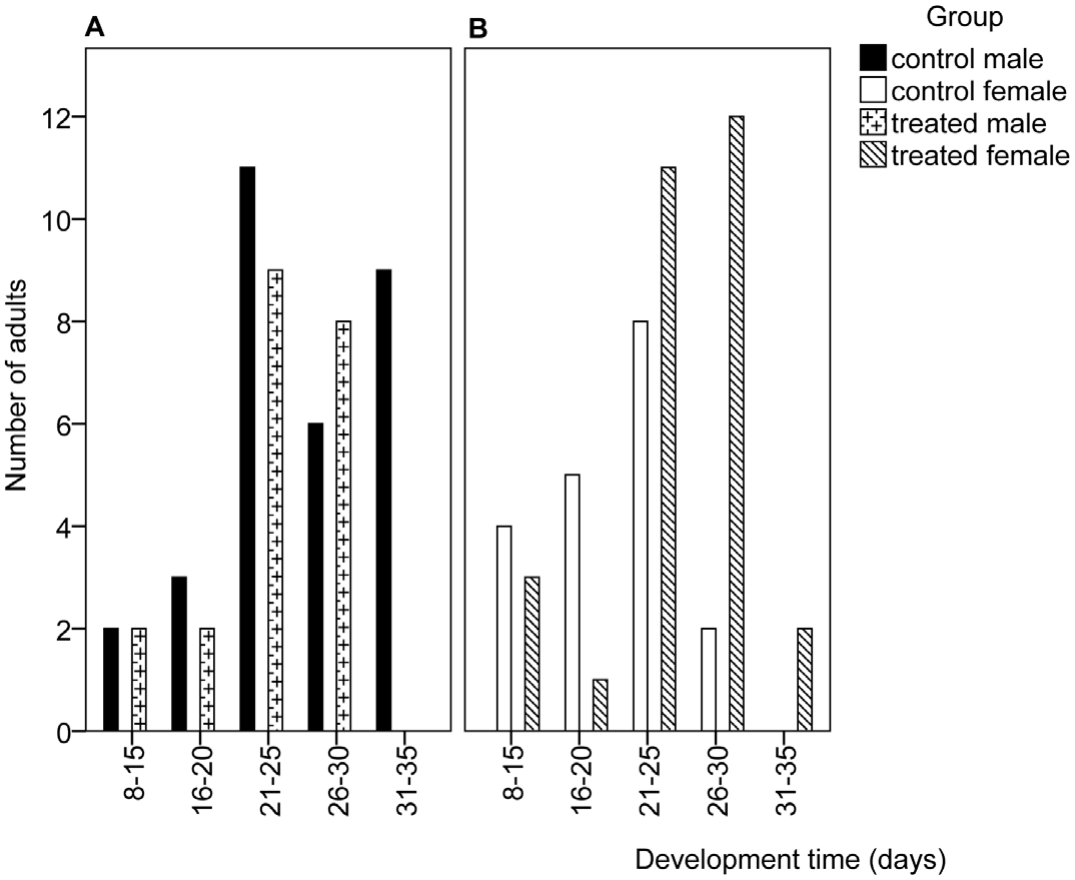
Range of development times for adults of *Nilaparvata lugens* after exposure as first instar nymphs at the ULT_50_. N = 50 for control and treated groups (gender ratio as in Figure 1).

The impact of exposure of first instar nymphs at the ULT_50_ temperature on development persisted into the adult stage; whilst the range of adult lifespans were again similar for treated females (8–31 days) and controls (13–30 days), the treated insects lived longer (F_1, 46_ = 5.950, p = 0.019, Figure 2B). However, treated males did not live as long as the control group (10–30 days and 14–35 days respectively, F_1, 50_ = 1.968, p = 0.167, Figure 2A).

### Effect of Sub-lethal High Temperatures on Fecundity

#### Treated nymphs vs treated adults

**Figure 3:**
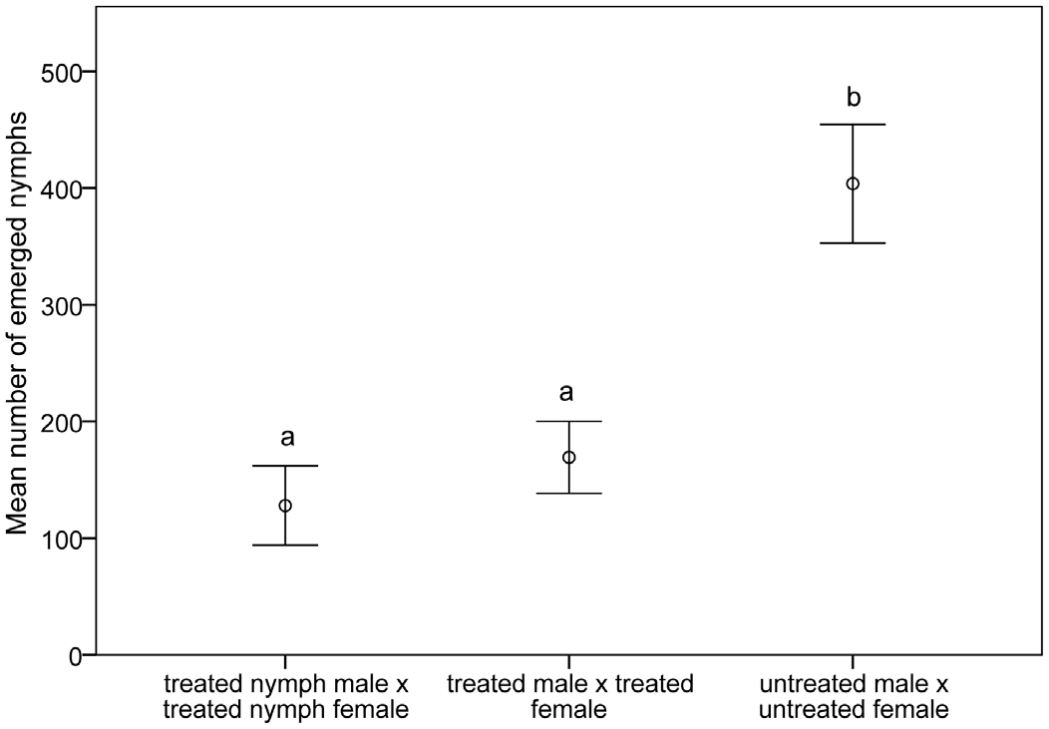
Mean number of eggs per female after exposure of first instar nymphs and adults of *Nilaparvata lugens* at their ULT_50_. N = 20 pairs for each mating combination. Mean values with the same letter are not significantly different at p<0.05 level.

After exposure at the ULT_50_ of 41.8 and 42.5°C at the first instar and adult stage respectively, mean egg production per female decreased from 403.8±13.7 in the untreated control to 128.0±16.6 (treated nymph male x treated nymph female) and 169.3±14.7 (treated male x treated female) (F_2, 57_ = 62.120, p<0.001, Figure 3), with a range of 267–627 eggs per female in the control, 34–317 in the treated nymph group and 84–326 in the treated adult group. Overall, mean egg production was most reduced when insects were exposed as first instar nymphs (31.7% of control group), than when both sexes were exposed as adults (reduction to 41.9% of control). However, there was no difference in mean egg production between treated nymph male x treated nymph female and treated male x treated female (p = 0.278).

#### Treated adult mating combinations

**Figure 4:**
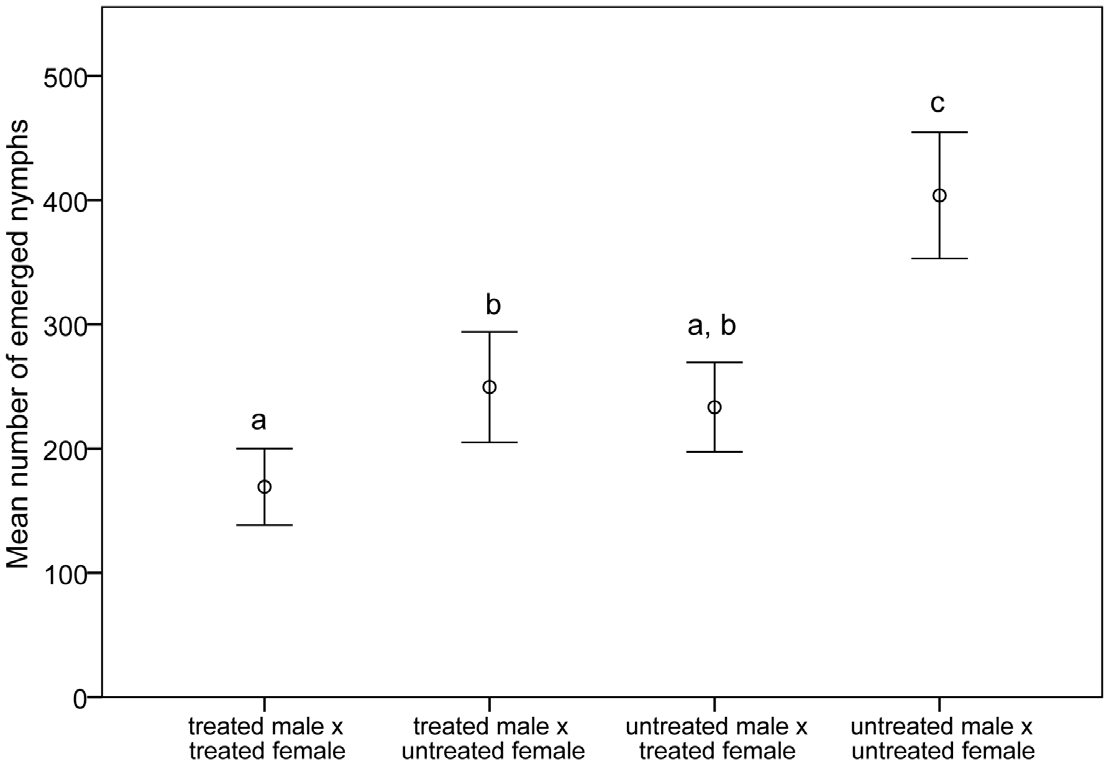
Mean number of eggs per female after exposure of adults of *Nilaparvata lugens* at their ULT_50_. N = 20 pairs for each mating combination. Mean values with the same letter are not significantly different at p<0.05 level.

For the three mating combinations after exposure of adults at the ULT_50_ of 42.5°C the mean number of eggs produced per female were: 169.3±14.7 (treated male x treated female, range 84–326), 249.6±21.3 (treated male x untreated female, range 75–436) and 233.4±17.2 (untreated male x treated female, range 94–412); F_3, 76_ = 25.470, with all adult mating combinations producing significantly fewer viable eggs than the control, p<0.001, Figure 4). Overall mean egg production was most reduced when both sexes had been exposed as adults (reduction to 41.9% of control), with less affect when only one sex was exposed as an adult (61.8% for treated male and 57.8% for treated female compared with the control group).

**Figure 5:**
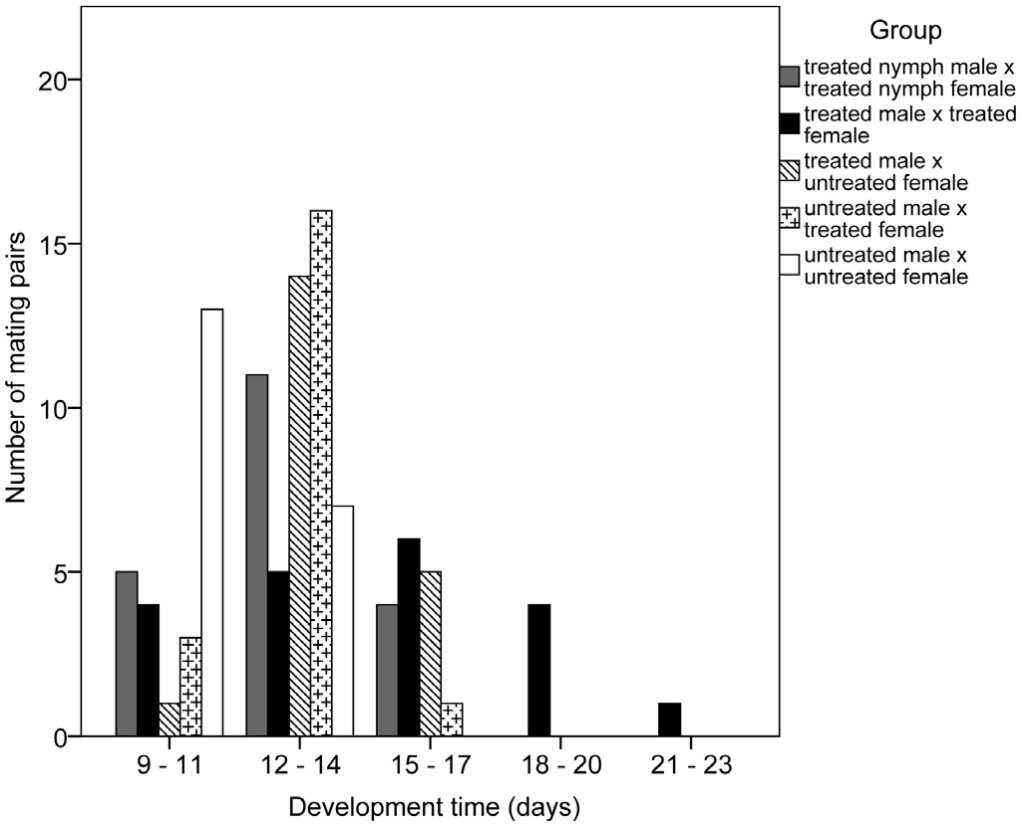
Range of egg development times after exposure of first instar nymphs and adults of *Nilaparvata* lugens at their ULT_50_. N = 20 pairs for each mating combination.

*Nilaparvata lugens* produced viable eggs in all mating groups that included insects exposed at their respective ULT_50_ temperatures (Figure 5). However, for all the treatment groups there was some delay until the first egg hatched and the range of egg development times was also extended in all the treated groups: 11–16 days for treated nymphs, 10–21 days for treated adult male and female, 11–16 days for treated male x untreated female, 10–16 days for untreated male x treated female, compared with 9–14 days in the control; all treated groups were significantly different to the control (F_4, 95_ = 10.616, p<0.001), but there was no difference between any of the treated groups.

## Discussion

Climate change operates on a global scale with wide-ranging and interrelated impacts across the social-economic-environmental interface ([94]). A greater understanding of the effects of climate warming on agricultural and natural ecosystems will inform policies aimed at mitigating risks, particularly with regard to ectothermic organisms for which temperature is an important determinant of development, survival and distribution ([54], [55], [95], [96], [97]). Insects have evolved a range of behavioural, physiological and biochemical adaptations to survive both seasonal and more acute fluctuations in temperature ([49]), but there are limits above and below which species cannot survive. A recent study with the brown plant hopper *Nilaparvata lugens* found that around 50% of first instar nymphs were killed by a brief exposure at 41.8°C (ULT_50_) and a similar proportion of adults at 42.5°C; both life cycle stages were immobilized by heat stress at lower temperatures ([28]). Whilst lethal temperatures provide estimates of the limits to survival, it cannot be assumed that individuals that survive at temperatures close to these limits are unaffected by the exposure ([98]) This study focused on the effects of sub-lethal high temperature exposure on the development and reproduction of *N. lugens*, a major pest of rice in tropical Asia.

After exposure of first instar nymphs at the ULT_50_ of 41.8°C development time to adult was significantly increased in both male and female *N. lugens*. The combination of nymphal development time and adult longevity resulted in an overall extension of the total life span of females but not males. A number of studies that have shown that males and females of several insect species differ in absolute performance capacities (e.g. consumption of resources, locomotor ability, duration of stress tolerance) when living under favourable (i.e. non-stressful) conditions ([48], [99], [100], [101]). As temperature is known to have a major influence on various ‘rate-based’ processes in ectotherms ([48]), the data suggest that there may be inherent differences in the thermal biology of males and females, or that they are differentially affected by exposure to high temperature. The results from this study also support the view that sub-lethal high temperatures can have a negative impact on insect development, especially at temperatures close to the upper thermal limit ([7], [102], [103]). The physiological explanation for impeded development following high temperature stress may be related to deleterious effects on respiratory metabolism ([104], [105], [106], [107], [108], [109]) or interference with the synthesis of hormones involved in the moulting process ([37], [110]).

As the eggs of *N. lugens* are laid in plant tissue, it is not possible to determine accurately the number of viable eggs laid, as some eggs would be destroyed when dissected out of the rice stems. Emergence of first instar nymphs was therefore used as an indicator of reproductive output. High temperature stress exerted a number of sub-lethal effects on reproduction in *N. lugens*: fewer nymphs emerged from eggs, the period of egg development was extended, and some nymphs were unable to moult to the second instar. An important factor that may contribute to the negative effects of high temperature stress on both development and reproduction in *N. lugens* concerns the role of the intracellular yeast-like symbiotes (YLS). In *N. lugens* and *L. striatellus* the YLS are contained in the fat body and transmitted transovarially between generations ([67]). The YLS are reported to play an important role in the abdominal segmentation and differentiation of planthopper embryos ([66]) and synthesise essential amino acids (that are vital for normal development) to compensate for variable amino acid availability in different plant hosts ([70]). Exposure of newly hatched nymphs of *L. striatellus* for 3 days at 35°C reduced the number of YLS by approximately 90% ([71]). The same treatment applied to nymphs of *N. lugens* for 3 days destroyed the YLS which in turn impeded development and ecdysis ([111]). Similarly, exposure at 32°C of 3 day-old adult females of *N. lugens* containing fully developed ovaries reduced the number of YLS and lowered fecundity ([64], [112]).

In a study on the pine false webworm *Acantholyda erythrocephala*, eggs failed to hatch at around 30°C ([113]). It is possible that the secretion of hormones from neurosecretory cells associated with egg production is inhibited by a direct heat exposure ([38]), but after transfer to favourable conditions, the reproductive activities are resumed in both males and females, but with a net reduction in overall fecundity. High temperature exposure may also reduce mating success, sperm viability and oviposition, all of which would impact negatively on generation-to-generation population abundance ([114], [115]). Also, whilst the effects of sub-lethal heat stress on *N. lugens* reported here arose from very brief exposures, in nature, the time periods involved would be much longer, unless the insects showed some form of avoidance behaviour. For example, large leaves of the host plants of *Manduca sexta* L. became hotter during the day than smaller leaves such that by selecting smaller leaves for oviposition, the thermal buffering of extreme temperatures would increase egg survival and successful hatching ([116]). A further consideration is that populations reared under laboratory conditions over long periods of time and multiple generations (with periodic refreshment with wild stock) may become increasingly different from natural populations through genetic bottlenecks ([117]). However, as population of *N. lugens* had been in culture for less than two years (and completed 11–12 generations), such effects are unlikely with the studied colony. It is also recognised that the effects of extreme exposures associated with climate change will most likely be revealed over longer term timescales and be subject to important interactions with other physical and biological factors ([118], [119]).

With these provisos in mind, the results from this study can be placed in a wider ecological context. Based on climatic data from various countries across the distribution of *N. lugens*, Piyaphongkul et al. ([28]) concluded that although mean temperatures were generally below the estimated ULT_50_ values of nymphs (41.8°) and adults (42.5°C) there were occasional extreme events that would overlap with these lethal temperatures, and that through heat-induced immobility at lower temperatures (at the CT_max_), insects may not be able to move away from potentially lethal exposure, or as has been identified in this study, deleterious effects of reproduction. When insects are heated (or cooled) at rates that are faster than those experienced in nature, the observed mortality (or other deleterious effects) may be caused by the range of temperatures experienced, the rate of change, the most extreme temperature experienced or a combination of all factors. When adult *N. lugens* were heated at 0.5°C min^−1^ to determine the ULT_50_ (42.5°C), no insects were killed until exposure at 42°C ([28]). As the same rate of warming was used in these experiments it seems reasonable to conclude that neither the change in temperature (approximately 20°) nor the rate of increase in temperature are detrimental to survival *per se* – rather, it is the highest temperature experienced that impedes development and lowers fecundity.

Across the distribution of *N. lugens* in tropical Asia there is considerable variation in winter minimum temperatures and also heat waves and more prolonged ‘hot spells’ in summer ([120]). Extreme temperatures of over 45°C occur over the north-west part of the region during May-June, and several countries in this region have reported increasing surface temperature trends in recent decades. For example, the annual mean surface air temperature in Vietnam, Sri Lanka and India has increased by 0.30–0.57°C per 100 years ([121]). Moreover, regional climate change simulations for the 21st century by Atmosphere-Ocean General Circulation Models (AOGCMs) relative to the baseline period of 1961–1990 suggest that the area-average annual mean surface air temperature over land areas of Asia will be higher by 1.6±0.2°C in the 2020s, 3.1±0.3°C in the 2050s and 4.6±0.4°C in the 2080s as a result of increases in the atmospheric concentration of greenhouse gas emissions ([121], [122]). Importantly, the influence of temperature on insect development is related not only to the daily or monthly mean values, but also to the rate of temperature change that will sometimes include extreme exposures ([103], [119]). Whilst the experiments reported here and the previous study on the lethal and behavioural thermal thresholds ([28]) suggest that *N. lugens* may be adversely affected across parts of its current distribution by high temperature stress and progressive climate warming, for some insects a warmer climate may be beneficial, as has been observed with the range expansion of the coffee berry borer (*Hypothenemus hampei*) ([123]). As such, the opportunity to benefit from a warmer climate (or not to suffer deleterious effects) lies in part in the difference in temperature between the upper lethal limit (and the range over which sub-lethal effects occur) and prevailing and future climatic regimes, and the ability to exploit new areas where necessary resources are available, but temperature has previously been a barrier to establishment and residency. Indeed, whilst Piyaphongkul et al. ([28]) highlighted areas where *N. lugens* might experience thermal stress under current climates, and would be more likely to do so in warmer climate (unless acclimation occurred), there were also parts of the distribution where winter low temperatures currently prevent year-round survival, but which might become more favourable through climate change.

In summary, the results reported here indicate that the temperatures that kill around 50% of nymphs and adults of *N. lugens* also exert negative effects on development and longevity. The same exposures also lower fecundity through a combination of effects that operate through both of the sexes, in which the greatest effects occur when both males and females have experienced sub-lethal heat stress.

## Materials and Methods

### Insect Materials

Adults of *N. lugens* were originally collected from the MARDI Research Station at Pulau Pinang in Malaysia. All insects in the stock culture and before and after experiments were reared on rice seedlings, *Oryza sativa* L. cv. TN 1, in cages or perspex boxes covered with 1.22 mm ventilation mesh at 16∶8 L:D and 23±0.5°C. Newly-hatched first-instar nymphs (within 48 h of hatching) and unmated adults (30–35 days old) were used in the experiments. All high temperature exposures were carried out in a programmable alcohol bath (Haake Phoenix 11 P2; Thermo Electron Corp., Germany) to an accuracy of ±0.5°C.

To investigate the effects of sub-lethal high temperature on development and fecundity of *N. lugens*, insects were exposed at their upper lethal temperature (ULT_50_). The ULT is determined by exposing insects at progessively higher temperatures and recording the mortality at each temperature. The ULT_50_ is the estimated temperature at which 50% of the population is killed ([25]).

### Effect of Sub-lethal High Temperatures on Development and Longevity

A sample of 150 newly-hatched first instar nymphs were warmed from 20°C at 0.5°C min^−1^ to their ULT_50_ (41.8°C), held for 2 min and then cooled at the same rate back to 20°C; preliminary experiments had indicated the time required for nymphs to be held at the ULT_50_ to experience the desired exposure temperature. When insects are heated or cooled, for example, in an alcohol bath, there is a time delay between the bath reaching the set temperature and the insects achieving thermal equilibrium at this temperature. This lag time is dependent on the thermal properties of the exposure system ([124]) and in general, larger insects will take longer to reach thermal equilibrium with the surrounding environment ([125], [126], [127], [128]).

From the surviving population a sample of 50 nymphs was placed individually on rice seedlings in Perspex boxes in the standard rearing conditions. A control group of 50 first instar nymphs were held individually in the same conditions. Daily observations were made to record the time taken to moult to adult and total longevity in the treatment and control groups. As the gender of the treated and untreated insects could not be determined at the first instar stage, the male and female sample sizes were not equal. A split-plot method was used to determine the main effects of treatment on the development and longevity of *N. lugens* using temperature treatment and sex as fixed factors in SPSS 17.0 software. In the split plot design, sex was a split plot factor within the temperature treatment.

### Effects of Sub-lethal High Temperatures on Fecundity

#### Nymphs

A sample 200 of newly-hatched first instar nymphs were heated from 20°C at 0.5°C min^−1^ to their ULT_50_ (41.8°C), held for 2 min, and then cooled back to 20°C at the same rate. Each surviving nymph was maintained individually in a Perspex rearing box containing a rice seedling. After moulting to adult, 20 treated females and males were randomly selected and transferred as pairs into separate rearing boxes with a rice seedling and maintained in the standard rearing conditions. Fecundity was measured by counting the number of emerging first instar nymphs at daily intervals until there was no further emergence.

#### Adults

A sample of 600 newly-hatched first-instar nymphs were reared together in a number of Perspex boxes containing rice seedlings until the late fifth instar, after which males and females were reared separately on rice seedlings to obtain unmated adults. For each mating combination, 100 adult virgin males and females were heated from 20°C at 0.5°C min^−1^ to their ULT_50_ (42.5°C), held for 6 min and then cooled back to 20°C at the same rate. From the surviving populations and a control population of the same age, 20 randomly selected pairs were established for each of three mating combinations: treated male x treated female, treated male x untreated female, and untreated male x treated female. The control group was created by allowing nymphs to develop from first to fifth instar after which the sexes were separated; 20 male and female pairs were taken from this stock and then allowed to mate and oviposit under the same conditions. Fecundity was measured in the same way as in the experiment with first instar nymphs.

All data were analysed by one-way analyses of variance (ANOVA) to test for the effect of treatment on the number of emerged nymphs between treated nymphs and treated adults, and among adult mating combinations. Where significant differences occurred, the data were further analysed using Tukey's honest significance difference post-hoc test and the Games-Howell test to separate statistically heterogenous and non-heterogenous groups respectively.
